# Seroprevalence, vaccination coverage and associated factors of hepatitis B and C infections among pregnant women in Azare, Bauchi State, Northeast Nigeria

**DOI:** 10.1186/s12879-026-13506-0

**Published:** 2026-05-04

**Authors:** Auwal Magaji, Muhammad Abdullahi, Zinat Mahmud, Sabiu Aminu, Amina Auwal

**Affiliations:** 1 Department of Microbiology, Federal University of Health Sciences, Azare, Bauchi State Nigeria; 2 Department of Public Health, National Open University of Nigeria, Lagos, Nigeria; 3 Department of Chemical Pathology, Federal University of Health Sciences, Azare, Bauchi State Nigeria; 4 Department of Biological Sciences, Federal University of Health Sciences Azare, Azare, Bauchi State Nigeria

**Keywords:** Antenatal care, Pregnant women, Hepatitis burden, Vaccination, Low education

## Abstract

**Background:**

Hepatitis B and C virus infections remain major public health challenges, particularly in low- and middle-income countries. Pregnant women are a high-risk group due to the potential for mother-to-child transmission. This study assessed the prevalence of HBV and HCV infections, vaccination coverage, and associated factors among pregnant women in Northeastern Nigeria.

**Methods:**

This hospital-based cross-sectional study was conducted between April and November 2025. Blood samples were collected from 423 pregnant women attending selected hospitals, after obtaining socio-demographic information. The samples were screened for HBsAg and anti-HCV antibodies using rapid diagnostic test kits.

**Results:**

The prevalence of HBV, HCV, and HBV/HCV co-infection were 9.93%, 4.49%, and 0.95%, respectively. Higher prevalence of HBV (4.96%; 95% CI: 2.89–7.03) and HCV (2.13%; 95% CI: 0.76–3.50) was observed among women aged 25–34 years. Vaccination coverage was relatively low and was significantly associated with educational level (*p* < 0.001) and place of residence (*p* < 0.001). Awareness and knowledge of hepatitis infection, transmission routes, and vaccine acceptance were significantly associated with vaccination uptake. The major barriers to vaccination included lack of awareness (42.32%), fear of side effects (22.93%), and misconceptions regarding vaccine necessity (20.57%).

**Conclusion:**

HBV and HCV infections remain prevalent among pregnant women in Northeastern Nigeria, with low vaccination coverage and significant gaps in awareness and access to preventive services, demanding targeted health education, improved vaccination access and strengthened antennal programs to reduce the burden of the infections in the area.

**Clinical trial number:**

Not applicable.

## Introduction

Hepatitis B and C virus infections are major global public health challenges, causing significant morbidity and mortality due to their progression to chronic liver disease, cirrhosis, and hepatocellular carcinoma (HCC) [1, 2]. In 2022, HBV and HCV infections accounted for an estimated 1.3 million deaths worldwide, primarily from cirrhosis and liver cancer [3]. Despite being preventable and treatable, both infections remain highly prevalent especially in low and middle income countries where access to vaccination, screening, and treatment services are limited [4]. Globally, an estimated 296 million and about 58 million people live with chronic HBV and HCV infections respectively [2, 5]. Sub-Saharan Africa bears a disproportionate burden, with HBV and HCV prevalence ranging between 6 and 12% and 1–4% respectively [6]. Nigeria has been reported to be among the most affected countries, with HBV and HCV prevalence of 9.5% and 1.4% respectively among general population [7]. Among pregnant women, HBV prevalence has been estimated to be 6–8%, with notable regional variations which may be driven by socioeconomic, cultural and healthcare disparities among the patients [8–10].

Regardless of the availability of highly effective HBV vaccines and curative direct-acting antivirals for HCV, major gaps remain in diagnosis, linkage to care, and treatment coverage, particularly among women of reproductive age [11, 12]. Despite increasing attention to HBV and HCV infections globally, evidence from many parts of sub-Saharan Africa, particularly Northeastern Nigeria, remains limited and fragmented. Most existing studies in Nigeria have focused either on hospital-based prevalence estimates or specific population groups, with little emphasis on pregnant women, attitudes, and vaccination practices that influence the transmission and control of the infections [13, 14]. This gap in contextual evidence limits the ability of public health authorities to design targeted interventions and vaccination campaigns among pregnant women in underserved settings. Furthermore, socioeconomic and behavioral factors that may influence awareness, perception of risk, and vaccine uptake are poorly documented in this region. Generating such evidence is essential for guiding effective prevention strategies and improving hepatitis control programs. Therefore, this study aimed to investigate the prevalence, vaccination coverage, and associated factors of HBV and HCV infections among pregnant women in Northeastern Nigeria, in order to provide evidence that can inform targeted public health interventions.

## Materials and methods

### Study design and setting

**Fig. 1:**
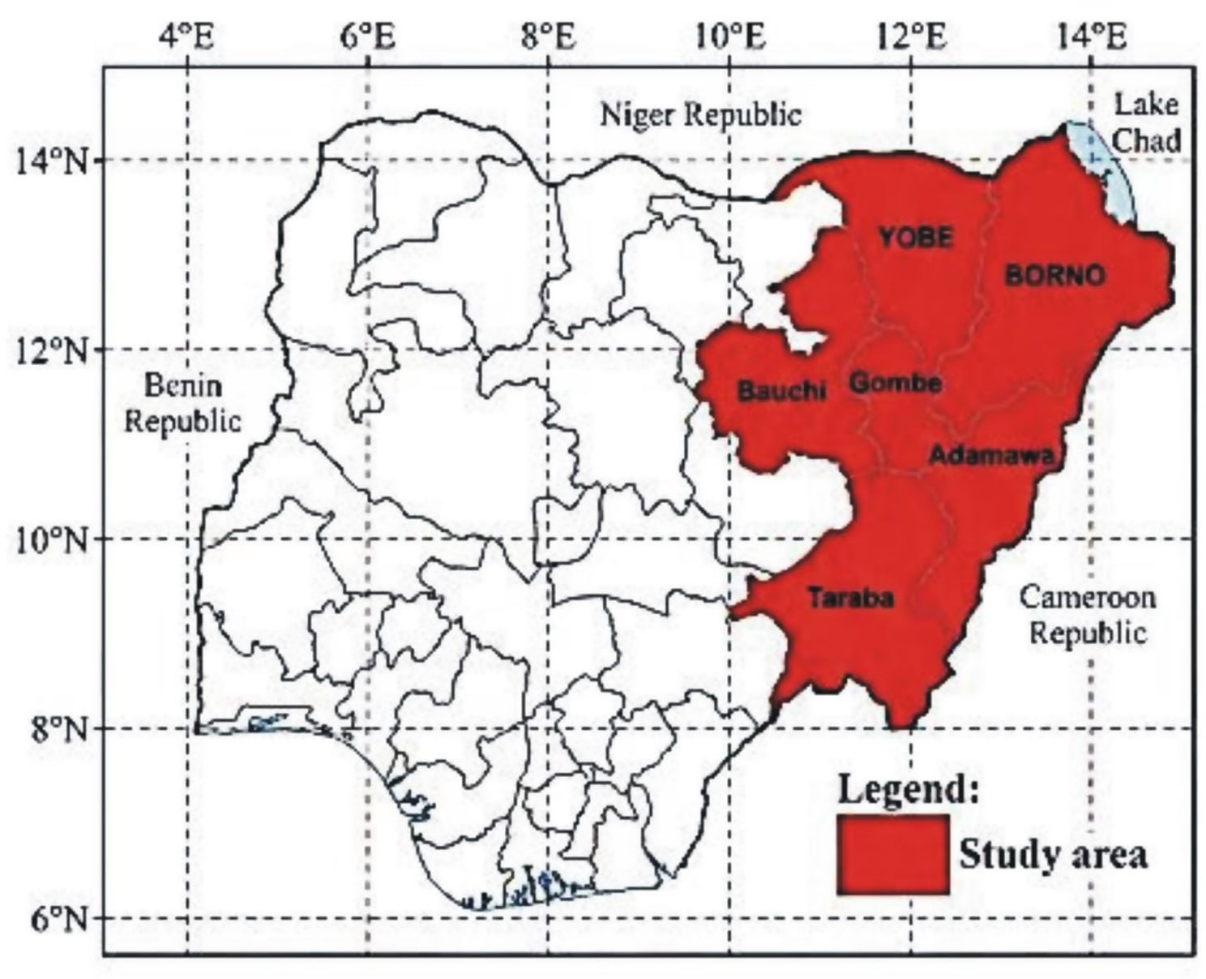
Map of Nigeria showing the study area

This hospital-based cross-sectional study was conducted at the Antenatal Clinics (ANC) of Extreme Hospital and General Hospital Azare, Bauchi state, Northeastern Nigeria between April and November 2025. The hospitals were selected because, they are among the major referral centers for antenatal care in the region, and attend to a large number of pregnant women from both urban and rural communities of Northeastern Nigeria (Fig. 1).

### Study population

The study population consisted of pregnant women attending antenatal care (ANC) at General Hospital and Extreme Hospital Azare during the study period. These two health facilities were selected because they are among the major healthcare centers providing antenatal services to a large population within Northeastern Nigerian. The sample size was proportionally allocated to the two hospitals based on the estimated average number of ANC attendees per clinic. Eligible participants were recruited using a consecutive sampling technique, where all pregnant women who attended the ANC clinic during each clinic day and consented to participate were enrolled until the required sample size was achieved.

All pregnant women who declined consent or critically ill and unable to respond to the questionnaires were excluded.

### Ethical considerations

Ethical approval was obtained from the Research Ethics Committee of Extreme Hospital (EXT25V002I) and General Hospital Azare (GHA/R33/1) before the start of the study. Written informed consent was obtained from each of the participants. Confidentiality was strictly maintained by using unique identifiers and secure storage of paper and electronic data. Participants who tested positive were counseled and referred to appropriate clinical services for further evaluation and management. All procedures were conducted in accordance with Declaration of Helsinki (2013).

### Sample size determination

Sample size was calculated using Cochran’s formula for estimating a proportion with specified precision as shown below:

$$\rm {n}=\:\frac{{{Z}\:}^{2}\times\:{P}(1-\:{P})}{{{d}}^{2}}$$

Where:

(Z) = standard normal deviate at 95% confidence level (1.96)

(p) = assumed prevalence = 0.50 (50%)

(d) = desired precision (margin of error) = 0.05

$$\rm Hence, n =\:\frac{{\left(1.96\right)}^{2}\:\times\:0.50(1\:-\:0.50)}{{0.05}^{2}}$$

$$\rm n = 385$$

To allow for non-response and unusable samples, a 10% contingency was added:

$$\rm n = 385 + 38.5$$

Therefore, the target sample size for this study was 423 pregnant women.

### Sampling technique

Consecutive sampling was used to recruit eligible pregnant women attending antenatal clinics during the study period until the required sample size (423) is reached. This method was adopted because it allows the inclusion of all eligible participants presenting during the study period.

### Data collection instruments and procedure

Structured questionnaires were developed after reviewing previously published studies assessing hepatitis knowledge, awareness, and vaccination practices among pregnant women and other at-risk populations. The questionnaires consisted of sections covering socio-demographic characteristics, awareness of hepatitis infection, knowledge of transmission and prevention, attitudes toward vaccination, and vaccination history. To ensure clarity and contextual relevance, the questionnaires were pretested among 20 pregnant women in a health facility outside the study area. Feedbacks from the pretests were used to modify ambiguous or unclear questions. The internal consistency of the knowledge and attitude items was assessed using Cronbach’s alpha reliability analysis, which yielded an acceptable reliability coefficient of ≥ 0.70, indicating adequate internal consistency of the instrument. In addition, the questionnaires were reviewed by Dr. M. M. Azare, an expert in epidemiology and public health to assess their content validity, ensuring that the items adequately captured the study variables.

### Operational definition of study variables

For the purpose of this study, the key variables related to HBV and HCV infections awareness, knowledge, attitudes, and vaccination status were defined and measured as follows:

1. Awareness of HBV and HCV infections: Awareness was assessed using a dichotomous question asking participants whether they had ever heard about HBV or HCV infection prior to the study. Participants who responded “Yes” were categorized as “aware”, while those who responded “No” were categorized as “not aware”.
2. Knowledge of HBV and HCV infections: Knowledge was assessed using a set of structured questions covering the causative agent, routes of transmission, and prevention methods of HBV and HCV infections. Each correct response was assigned a score of “1” standing for “know”, while incorrect or “don’t know” responses were assigned “0”. The scores were summed to obtain an overall knowledge score for each participant. Participants who correctly answered at least 50% of the knowledge questions were categorized as having “good knowledge”, while those scoring below this threshold were categorized as having “poor knowledge”. This is adopted from previous studies assessing hepatitis knowledge among study participants.
3. Attitude toward HBV and HCV infections prevention: Attitude was evaluated using questions assessing participants’ beliefs regarding whether HBV and HCV infections can be prevented and their perception of vaccination as a preventive measure. Responses were categorized into “Yes” (belief that hepatitis can be prevented and vaccination is beneficial) and “No” (belief that hepatitis cannot be prevented or skepticism toward vaccination).
4. Vaccination status: Vaccination status was determined based on self-reported history of HBV vaccination. Participants who reported receiving at least one dose of the HBV vaccine were classified as “vaccinated”, while those who reported no prior vaccination were classified as “not vaccinated”.
5. Vaccine acceptance: Vaccine acceptance was assessed by asking participants whether they would be willing to receive the HBV vaccine if it were available. Participants answering “Yes” were categorized as “willing to accept vaccination”, while those answering “No” were categorized as “not willing”.

### Specimen collection and laboratory testing

Approximately 5 mL of venous blood was collected aseptically from each participant into plain tubes. The samples were properly labeled and transported to the laboratory of each of the selected hospitals for HBsAg and anti-HCV antibodies screening.

The blood samples collected were centrifuged at 3000 rpm for 10 min to separate the serum. The obtained serum samples were used for the detection of hepatitis B surface antigen (HBsAg) and hepatitis C virus antibodies (anti-HCV) using rapid diagnostic test kits (Bio Rad, France), in accordance with the World Health Organization (WHO) guidelines for hepatitis B and C testing and the manufacturers’ instructions. Confirmation of the positive samples was done using Enzyme-linked Immunosorbent Assay (ELISA) (Bio Rad, France) [15].

Approximately 150 µL of serum from each participant were dispensed into the sample well of the test device for both HBsAg and anti-HCV kits. Subsequently, 100 µL of the assay buffer supplied with the kits were added to facilitate the chromatographic reaction. The test devices were allowed to stand at room temperature for 20 min before reading the results.

The test results were interpreted according to the manufacturer’s instructions and WHO recommendations. The appearance of two distinct red lines, one in the control region (C) and another in the test region (T), indicated a positive result for either HBsAg or anti-HCV. A single red line in the control region (C) indicated a negative result, suggesting the absence of detectable HBsAg or anti-HCV antibodies in the sample. Tests were considered invalid if no line appeared in the control region, and such tests were repeated [16].

The rapid diagnostic test kits used for the detection of hepatitis B surface antigen (HBsAg) and anti-HCV antibodies were manufactured by Bio-Rad (France) and used according to the manufacturer’s instructions and WHO recommendations. According to the manufacturer’s specifications, the HBsAg rapid test has a reported sensitivity of approximately 99.0% and specificity of 99.5%, while the anti-HCV antibody test has a sensitivity of approximately 98.0% and specificity of 99.0%. To further improve diagnostic accuracy, all positive rapid test results were confirmed using enzyme-linked immunosorbent assay (ELISA).

### Laboratory quality control

To ensure the reliability and accuracy of laboratory results, all tests were conducted strictly according to the manufacturer’s instructions. Positive and negative control samples supplied with the test kits were used to validate the performance of the assays before testing participant samples.

### Data handling procedure

Completed questionnaires were checked for completeness and consistency. Data were coded and entered into Microsoft Excel before being exported to SPSS v23.0 for analysis. Data cleaning was conducted to identify missing values and inconsistencies prior to statistical analysis. Questionnaires with substantial missing information were excluded from the analysis. For variables with minor missing responses, analyses were conducted using complete case analysis, where only observations with available data for the variables of interest were included. The proportion of missing data was minimal and therefore unlikely to significantly influence the study findings.

### Data management and statistical analysis

Data were entered into Microsoft Excel and analyzed using SPSS version 23.0 (IBM Corp., Armonk, NY, USA). Descriptive statistics such as frequencies and percentages were used to summarize the data. The association between categorical variables and HBV and HCV infections was initially assessed using the chi-square test. Variables with a p-value ≤ 0.20 in the bivariate analysis were included in a multivariable logistic regression model to identify independent predictors of the infections and control for potential confounding variables. Adjusted odds ratios (AOR) with 95% confidence intervals were also calculated. A *p*-value < 0.05 was considered statistically significant.

In addition to statistical significance in the bivariate analysis (*p* ≤ 0.20), variables were selected for inclusion in the multivariable logistic regression model based on their epidemiological relevance and evidence from previous literature suggesting their association with hepatitis vaccination behavior and infection risk. This approach ensured that potential confounders such as residence, awareness, knowledge, and health-seeking behaviors were not excluded solely on the basis of statistical thresholds.

Also, prior to multivariable analysis, multicollinearity among independent variables was assessed using the Variance Inflation Factor (VIF). Variables with VIF values greater than 5 were considered indicative of potential multicollinearity and were carefully evaluated before inclusion in the final model.

The goodness-of-fit of the logistic regression model was evaluated using the Hosmer–Lemeshow test, where a non-significant p-value indicated an adequate model fit. Model stability and plausibility of estimates were also assessed by examining standard errors and confidence intervals.

## Results

### Prevalence of HBV and HCV infections in the study area

**Fig. 2:**
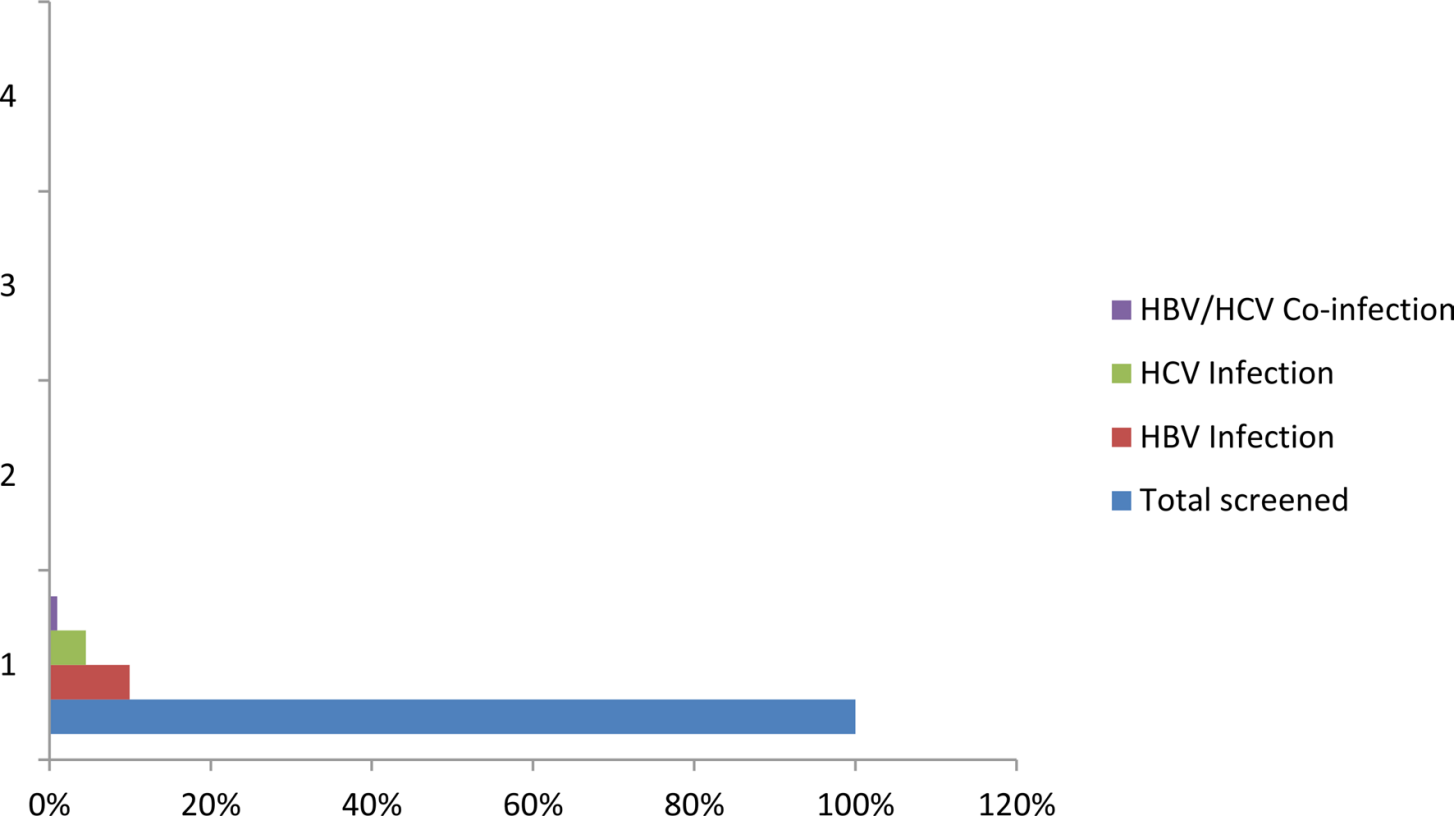
Overall prevalence of HBV and HCV infections among the study participants

The present study revealed that, out of the total 423 pregnant women enrolled in the study, 42 tested positive for HBV infection, 19 for HCV infection, and 4 for HBV/HCV co-infection, giving overall prevalence values of 9.93%, 4.49% and 0.95% respectively. Prevalence estimates were calculated using the total number of participants screened (*n* = 423) as the denominator (Fig. 2).

With respect to age, the current study showed that, participants aged 25–34 years had the highest prevalence of both HBV infection 4.96% (95% CI: 2.89–7.03) and HCV infection 2.13% (95% CI: 0.76–3.50). Participants aged 35–44 years showed moderate prevalence levels for HBV infection 2.84% (95% CI: 1.25–4.43) and HCV infection 1.65% (95% CI: 0.43–2.87), while those aged 15–24 years had relatively lower prevalence of HBV infection 2.13% (95% CI: 0.76–3.50) and HCV infection 0.71% (95% CI: 0.00-1.51), but none of the women aged 45 years and above was found positive. These prevalence estimates were calculated using the total study population (*N* = 423) as denominator (Table 1).

Out of the total study population (*N* = 423), participants with no formal education had the highest prevalence of HBV infection 4.02% (95% CI: 2.16–5.90) and HCV infection 2.13% (95% CI: 0.76–3.50), whereas those with tertiary education had the lowest prevalence of HBV infection 0.95% (95% CI: 0.02–1.88) and HCV infection 0.47% (95% CI: 0.00-1.11) (Table 1).

Regarding place of residence, participants living in rural areas had higher prevalence of both HBV infection 5.67% (95% CI: 3.46–7.88) and HCV infection 2.60% (95% CI: 1.08–4.12) compared to those living in urban areas, where HBV and HCV infections prevalence were 4.26% (95% CI: 2.34–6.18) and 1.89% (95% CI: 0.60–3.18) respectively. For this prevalence estimate, 423 was also used as denominator (Table 1).

**Table 1:** Distribution and prevalence of HBV and HCV infections according to demographic characteristics of the participants

Variables		HBV Positive	HBV Prevalence (%)	HCV Positive	HCV Prevalence (%)
Age group					
	15–24 years	9	2.13 (95% CI: 0.76–3.50)	3	0.71 (95% CI: 0.00-1.51)
	25–34 years	21	4.96 (95% CI: 2.89–7.03)	9	2.13 (95% CI: 0.76–3.50)
	35–44 years	12	2.84 (95% CI: 1.25–4.43)	7	1.65 (95% CI: 0.43–2.87)
	> 45 years	0	0.00	0	0.00
Education Level					
	None	17	4.02 (95% CI: 2.16–5.90)	9	2.13 (95% CI: 0.76–3.50)
	Primary	13	3.07 (95% CI: 1.43–4.71)	5	1.18 (95% CI: 0.14–2.22)
	Secondary	8	1.89 (95% CI: 0.60–3.18)	3	0.71 (95% CI: 0.00-1.51)
	Tertiary	4	0.95 (95% CI: 0.02–1.88)	2	0.47 (95% CI: 0.00-1.11)
Residence					
	Urban	18	4.26 (95% CI: 2.34–6.18)	8	1.89 (95% CI: 0.60–3.18)
	Rural	24	5.67 (95% CI: 3.46–7.88)	11	2.60 (95% CI: 1.08–4.12)

Prevalence percentages were calculated using the total number of participants (*N* = 423) as denominator

### Hepatitis vaccination coverage by demographic characteristics of the participants

The current study revealed that, the association between age and vaccination status was not statistically significant (χ² = 2.613, *p* = 0.455). In contrast, education level showed a strong and statistically significant association with vaccination status (χ² = 183.534, *p* < 0.001). Participants with tertiary education had the highest vaccination coverage, whereas those with no formal education had relatively low vaccination uptake (Table 2).

Place of residence also showed a significant association with vaccination status (χ² = 15.636, *p* < 0.001). Urban residents had higher vaccination coverage (23.40%) compared with rural residents (20.09%) (Table 2).

**Table 2:** Hepatitis vaccination coverage across demographic characteristics of the participants

Demographic factors	Vaccinated	Not Vaccinated	χ²	df	*P* value
**Age Group**					
15–24 **years**	23 (5.44%)	27 (6.38%)	2.613	3	0.455*
25–34 **years**	91 (21.51%)	113 (26.71%)			
35–44 **years**	50 (11.82%)	60 (14.18%)			
45 **years** and above	20 (4.73%)	39 (9.22%)			
**Education Level**					
No Formal Education	1 (0.24%)	77 (18.20%)	183.534	3	0.000*
Primary Education	11 (2.60%)	27 (6.38%)			
Secondary Education	23 (5.44%)	95 (22.46%)			
Tertiary Education	149 (35.22%)	40 (9.46%)			
**Residence**					
Urban	99 (23.40%)	173 (40.90%)	15.636	1	0.000*
Rural	85 (20.09%)	66 (15.60%)			

χ² = Chi-square

df = degree of freedom

‘*’ = significant at *p* < 0.05

### Logistic regression analysis of factors associated with hepatitis vaccination status among the participants

Binary logistic regression analysis showed that, awareness of HBV and HCV infections (COR = 1.81; 95% CI: 1.07–3.07; *p* = 0.028), knowledge of the causative agent (COR = 2.04; 95% CI: 1.33–3.15; *p* = 0.001), willingness to accept vaccination (COR = 7.65; 95% CI: 3.20–18.30; *p* < 0.001), and willingness to undergo hepatitis screening (COR = 6.51; 95% CI: 2.71–15.66; *p* < 0.001) were significantly associated with vaccination status. Participants who believed that hepatitis can be prevented had substantially higher odds of vaccination (COR = 48.41; 95% CI: 6.62–354.13; *p* < 0.001). Urban residence was also significantly associated with vaccination uptake (COR = 2.25; 95% CI: 1.50–3.38; *p* < 0.001). Although those with prevention knowledge had higher vaccination odds (COR = 1.90; 95% CI: 0.81–4.44; *p* = 0.139), the association was not statistically significant (Table 3).

Multivariable logistic regression analysis showed that knowledge of the causative agent (AOR = 1.76; 95% CI: 1.10–2.83; *p* = 0.019), belief that HBV and HCV infections can be prevented (AOR = 21.63; 95% CI: 2.84–164.73; *p* = 0.003), willingness to accept vaccination (AOR = 4.92; 95% CI: 2.01–12.06; *p* < 0.001), and willingness to undergo hepatitis screening (AOR = 3.8; 95% CI: 1.57–9.38; *p* = 0.003) were significantly associated with vaccination status. Urban residence remained significantly associated with vaccination uptake after adjusting for other variables (AOR = 1.68; 95% CI: 1.08–2.61; *p* = 0.021) (Table 3).

**Table 3:** Binary and multivariable logistic regression analyses of factors associated with hepatitis vaccination status among the participants

Variables	Vaccinated	COR	*P* value	AOR	*P* value
**Awareness**					
Heard about hepatitis infection	160 (37.83%)	1.81 (95% CI: 1.07–3.07)	0.028*	1.42 (95% CI: 0.82–2.45)	0.205
Not heard	24 (5.67%)	1		1	
**Knowledge of the Infection Origin**					
Know the causative agent	142 (33.57%)	2.04 (95% CI: 1.33–3.15)	0.001*	1.76 (95% CI: 1.10–2.83)	0.019*
Don’t know	42 (9.93%)	1		1	
**Knowledge of Transmission Routes**					
Know	104 (24.59%)	0.53 (95% CI: 0.35–0.79)	0.002*	0.71 (95% CI: 0.44–1.15)	0.162
Don’t know	80 (18.91%)	1		1	
**Prevention Knowledge**					
Know	176 (41.61%)	1.90 (95% CI: 0.81–4.44)	0.139	1.36 (95% CI: 0.55–3.35)	0.504
Don’t know	8 (1.89%)	1		1	
**Attitude towards Prevention**					
Can be prevented	183(43.26)	48.41 (95% CI: 6.62–354.13)	< 0.001*	21.63 (95% CI: 2.84–164.73)	0.003*
Cannot be prevented	1 (0.24%)	1		1	
**Vaccine Acceptance**					
Will accept	178 (42.08%)	7.65 (95% CI: 3.20–18.30)	< 0.001*	4.92 (95% CI: 2.01–12.06)	< 0.001*
Will not accept	6 (1.42%)	1		1	
**Health-Seeking Behavior**					
Willing for hepatitis screening	178 (42.08%)	6.51 (95% CI: 2.71–15.66)	< 0.001*	3.84 (95% CI: 1.57–9.38)	0.003*
Not willing	6 (1.42%)	1		1	
**Residence**					
Urban	99 (23.40%)	2.25 (95% CI: 1.50–3.38)	< 0.001*	1.68 (95% CI: 1.08–2.61)	0.021*
Rural	85 (20.09%)	1		1	

*Statistically significant variables: *p* < 0.05

### Barriers to HBV/HCV vaccination among the study participants

**Fig. 3:**
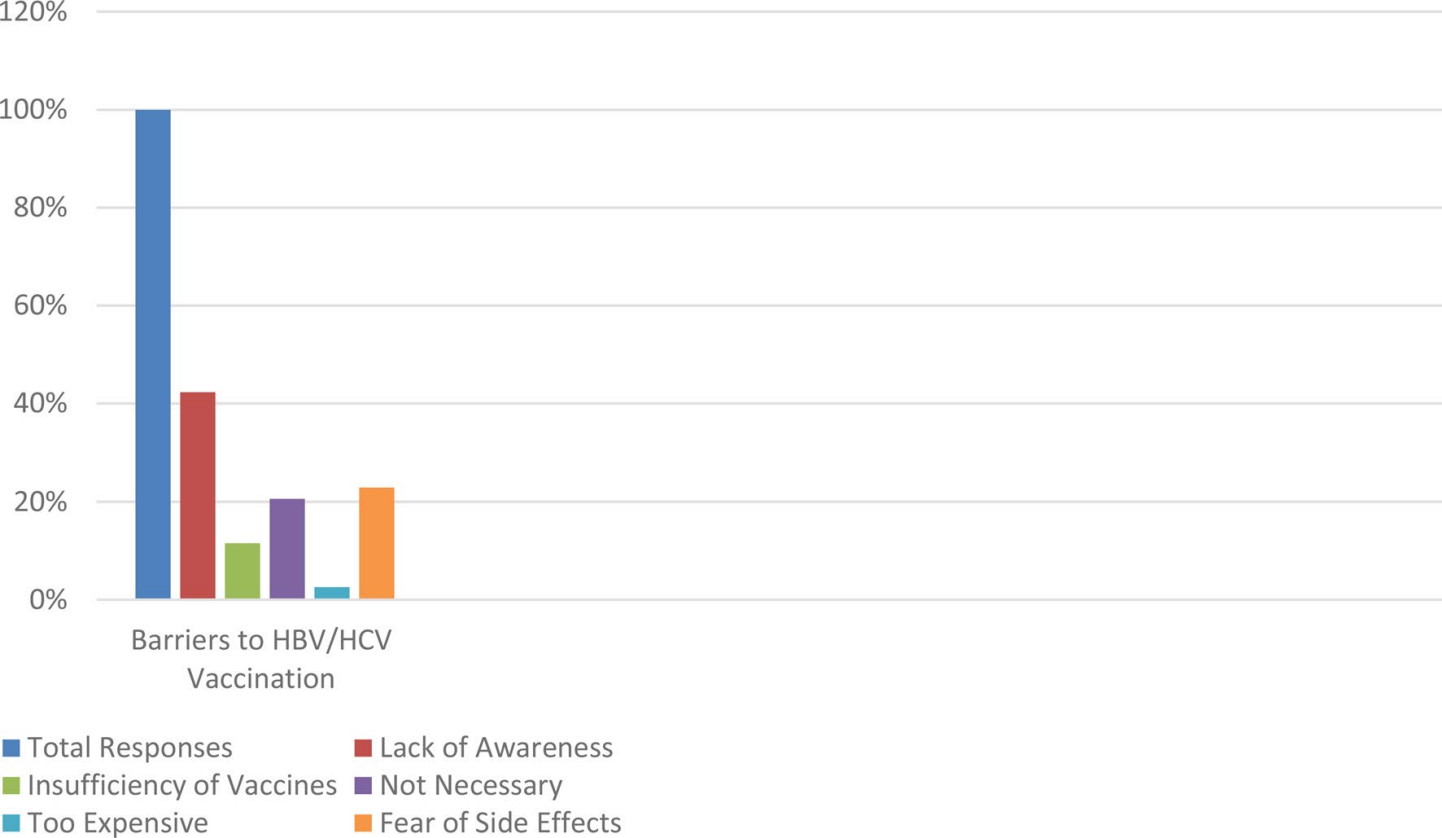
Barriers to HBV/HCV vaccination among the study participants

Of all the participants, 42.32% reported lack of awareness regarding the vaccination. Scarcity or lack of hepatitis vaccines in some areas particularly rural settings has also been reported by 11.58% of the participants. Also, 20.57% perceived that, vaccination is not necessary in preventing hepatitis. Similarly, high cost of vaccines in some parts of the study area prevented 2.60% of the participants from being vaccinated. About, 22.93% of the participants were not vaccinated as a result of fear of side effects of the vaccines (Fig. 3).

## Discussion

The present study assessed the prevalence, vaccination coverage, and associated factors of HBV and HCV infections among pregnant women attending antenatal care at Extreme Hospital and General Hospital Azare, Bauchi State, Northeastern Nigeria. The findings showed that HBV infection remains relatively common among the study population, with a prevalence of 9.93%, while HCV prevalence was 4.49%. These findings highlight the continued burden of viral hepatitis among pregnant women in the region and reinforce the importance of strengthening preventive and screening strategies within antenatal care services.

The prevalence of HBV observed in this study is comparable to several reports from other parts of Nigeria and sub-Saharan Africa. For example, similar prevalence levels have been reported in Nasarawa State (8.3%), Kogi State (10.6%), and other regions of Nigeria (9.4%) [7, 17–20]. However, the prevalence is slightly higher than those reported in Lafia (8.0%), Otukpo (5.6%), and Gambella, Ethiopia (7.9%) [21–25].

These differences may be associated with variations in study populations, geographical locations, cultural practices, and access to healthcare services. Differences in screening methods and sample sizes may also contribute to variations in reported prevalence across studies.

The prevalence of HCV infection observed in this study was lower than that of HBV but remains epidemiologically relevant. This pattern is consistent with the general epidemiology of viral hepatitis in sub-Saharan Africa, where HBV infection is typically more prevalent than HCV infection [26]. The presence of HBV/HCV co-infection among a small proportion of participants (0.95%) is noteworthy because co-infection has been associated with accelerated disease progression and may complicate clinical management. Although the proportion of co-infected participants was relatively small in this study, the finding underscores the importance of routine screening for both infections during pregnancy.

Age-related patterns in HBV and HCV infections prevalence were also observed. Women aged 25–34 years had the highest prevalence of both HBV (4.96%; 95% CI: 2.89–7.03) and HCV (2.13%; 95% CI: 0.76–3.50) infections. Similar age-related trends have been reported in other studies conducted in Nigeria and elsewhere, where infections prevalence tend to be higher among participants in their reproductive and economically active years [19]. This pattern may reflect differences in exposure to potential risk factors, including sexual activity, healthcare procedures, and cumulative lifetime exposure to infection sources. However, because the present study used a cross-sectional design, the observed relationship between age and infection prevalence should be interpreted as an association rather than a causal relationship.

Educational status showed a clear relationship with HBV and HCV infections prevalence in this study. Women with no formal education had the highest prevalence of both HBV 4.02% (95% CI: 2.16–5.90) and HCV 2.13% (95% CI: 0.76–3.50) infections, whereas those with tertiary education had the lowest prevalence. This pattern is consistent with findings from other studies conducted in Nigeria, which suggest that educational attainment may reflect differences in awareness, health literacy, and access to healthcare services [19, 27]. Women with higher educational levels may have greater access to health information and preventive services, which could influence their exposure to infection risk factors and adoption of preventive behaviors.

Place of residence was also associated with differences in the infections prevalence. Participants residing in rural areas showed higher prevalence of both HBV 5.67% (95% CI: 3.46–7.88) and HCV 2.60% (95% CI: 1.08–4.12) infections compared with those living in urban areas. This pattern may reflect disparities in access to healthcare services, vaccination programs, and health education initiatives between rural and urban communities [27]. Rural populations may face barriers such as limited healthcare infrastructure, reduced availability of screening services, and lower levels of health awareness, which could contribute to the observed differences in the HBV and HCV infections prevalence.

Vaccination coverage in the study population was relatively low, particularly among women with no formal education and those residing in rural areas. The significant association observed between educational status and vaccination uptake (*p* < 0.001), suggests that educational attainment may be associated with preventive health behaviors, including vaccine utilization. Similarly, the higher vaccination coverage (23.40%) observed among urban residents may reflect better access to healthcare facilities and immunization services in urban settings. These findings are consistent with previous studies that reported low vaccination uptake in developing countries despite the availability of effective vaccines [27, 28]. These highlight potential inequalities in access to vaccination services that may exist between different population groups.

The study also examined behavioral and knowledge-related factors associated with hepatitis vaccination status. Awareness of hepatitis infection, knowledge of the causative agent, and willingness to accept vaccination were significantly associated with vaccination uptake in the regression analysis. Women who demonstrated knowledge of the causative agent were more likely to be vaccinated compared with those who lacked such knowledge. These findings suggest that improved knowledge and awareness of hepatitis infection may be linked to increased uptake of preventive measures such as vaccination. These findings are supported by several earlier studies that clearly reported the association between knowledge of the infections and vaccination uptake [19, 27].

Attitudes toward hepatitis prevention also showed a strong association with vaccination status. Participants who believed that hepatitis infection can be prevented were substantially more likely to be vaccinated than those who did not share this belief. However, the very large odds ratio observed for this variable, accompanied by a wide confidence interval (AOR = 21.63; 95% CI: 2.84–164.73; *p* = 0.003), should be interpreted with caution. This may reflect the small number of participants who reported negative attitudes toward prevention, which can produce unstable estimates in regression analysis. Nevertheless, the finding suggests that perceptions regarding disease preventability may play an important role in shaping preventive health behaviors.

In addition to knowledge and attitudes, health-seeking behaviors were associated with vaccination uptake. Participants who expressed willingness to undergo hepatitis screening were significantly more likely to be vaccinated. This finding may indicate that individuals who demonstrate proactive health-seeking behaviors are also more inclined to adopt preventive health measures. Such patterns have been observed in other public health studies where health awareness and engagement with healthcare services are linked to improved preventive practices [29].

Several barriers to hepatitis vaccination were identified among the participants. Lack of awareness was the most commonly reported barrier, followed by fear of side effects and misconceptions regarding the necessity of vaccination. These findings highlight the presence of information gaps and potential misconceptions about hepatitis vaccination within the study population. Addressing these barriers through targeted health education and community engagement strategies may therefore be important for improving vaccination coverage. These are supported by several previous studies that reported similar trends [28, 30, 31].

The findings of this study have important implications for HVB and HCV infections prevention strategies in Northeastern Nigeria. The relatively high prevalence of HBV and the low vaccination coverage observed among pregnant women suggest that antenatal care services could serve as a critical platform for strengthening the infections prevention efforts. Integrating routine hepatitis screening, vaccination counseling, and health education into antenatal care programs may help improve early detection and preventive practices among pregnant women. Furthermore, the disparities observed between rural and urban populations indicate the need for targeted interventions that improve access to vaccination and screening services in underserved rural communities.

Although several factors were significantly associated with vaccination uptake and infection prevalence, the cross-sectional nature of the study does not allow causal relationships to be established. Nevertheless, the findings provide important evidence on the epidemiology of HVB and HCV infections and vaccination behaviors among pregnant women in Northeastern Nigeria and may inform future public health interventions aimed at reducing the burden of viral hepatitis in the region.

### Strengths and limitations of the study

This study provides important insights into the burden of HVB and HCV infections among pregnant women attending antenatal clinics in Northeastern Nigeria. One of the major strengths of the study is the relatively large sample size of 423 participants, which improves the reliability of the prevalence estimates. In addition, the study simultaneously assessed infection prevalence, vaccination coverage, and behavioral factors such as awareness, knowledge, and attitudes toward hepatitis infection, providing a comprehensive understanding of the epidemiological and behavioral determinants of hepatitis among pregnant women. The use of rapid diagnostic test kits with ELISA confirmation further strengthened the validity of the laboratory findings.

However, several limitations should be acknowledged. First, the study was conducted among pregnant women attending antenatal care in two hospitals, which may introduce selection bias, as women who attend ANC services may differ from those who do not seek antenatal care in terms of socioeconomic status, health awareness, and access to healthcare. Therefore, the findings may not be fully generalizable to all pregnant women in the wider community.

Secondly, some variables were based on self-reported information, such as vaccination history, awareness, and health-seeking behaviors, which may be subject to recall bias or social desirability bias. Participants may have inaccurately reported their vaccination status or knowledge levels.

Thirdly, although rapid diagnostic tests were used for initial screening and ELISA for confirmation, diagnostic limitations may still exist, including the possibility of false-negative or false-positive results. In addition, molecular techniques such as polymerase chain reaction (PCR), which could provide more accurate detection of viral infection and viral load, were not performed due to resource constraints.

Fourthly, although multivariable logistic regression was used to adjust for potential confounding variables, residual confounding cannot be completely excluded because not all possible risk factors (such as sexual behavior, prior blood transfusion history, or detailed medical exposures) were captured in the questionnaire.

Finally, the logistic regression model may be subject to model instability or overfitting, particularly for variables with small subgroup sample sizes, which may partly explain the large odds ratios and wide confidence intervals observed for some predictors. Therefore, these estimates should be interpreted cautiously.

Despite these limitations, the study provides valuable baseline data on HVB and HCV prevalence, vaccination coverage, and associated factors among pregnant women in Northeastern Nigeria, which can inform targeted public health interventions and future research.

## Conclusion

HBV and HCV infections are still common among pregnant women in Northeast Nigeria, with low vaccination coverage and significant gaps in awareness. Strengthening prenatal screening, improving access to vaccines, and implementing targeted health education may contribute to reducing the burden of hepatitis infections and improving prevention among pregnant women in the region.

## Recommendations

Based on the findings of this study, the following recommendations are proposed:

1. Strengthening routine hepatitis screening in antenatal care: given the relatively high prevalence of HBV and HCV observed among pregnant women in this study, routine screening for HVB and HCV should be consistently implemented and strengthened in antenatal clinics to facilitate early diagnosis and reduce the risk of mother-to-child transmission.
2. Expansion of hepatitis B vaccination services: because vaccination coverage was found to be significantly associated with educational level and place of residence, hepatitis vaccination services should be expanded particularly in rural communities and primary healthcare facilities where access may be limited.
3. Targeted health education interventions: the study showed that awareness and knowledge of hepatitis infection were significantly associated with vaccination uptake. Therefore, structured health education programs should be integrated into antenatal care services to improve understanding of hepatitis transmission, prevention, and the benefits of vaccination.
4. Addressing misconceptions and vaccine hesitancy: since a considerable proportion of participants reported fear of side effects and misconceptions about vaccine necessity, public health campaigns should focus on correcting misinformation and improving community trust in vaccination programs through engagement with healthcare workers, community leaders, and local media.
5. Further research: future studies using community-based and longitudinal designs are recommended to better understand transmission patterns and evaluate the effectiveness of intervention strategies aimed at improving hepatitis prevention among pregnant women.

## Data Availability

All data for this study are contained in the manuscript. Additional information and materials are obtainable from the corresponding author on a reasonable request.

## Abbreviations

ANC: Antenatal Care
CI: Confidence Interval
Df: Degrees of Freedom
HBsAg: Hepatitis B surface antigen
HBV: Hepatitis B virus
HCC: Hepatocellular carcinoma
HCV: Hepatitis C virus
RDT: Rapid diagnostic test
Rpm: Revolutions per minute
SPSS: Statistical Package for the Social Sciences
